# A rare giant extraocular, anterior chest wall sebaceous carcinoma

**DOI:** 10.1016/j.ijscr.2019.11.054

**Published:** 2019-12-03

**Authors:** Joseph Motshedi Sekgololo, Risenga Frank Chauke, Nonkutalo Tshazi

**Affiliations:** a Cardiothoracic Surgery Department, Sefako Makgatho Health Sciences University, Dr George Mukhari Academic Hospital, Pretoria, South Africa; b Plastic Surgery Department, Sefako Makgatho Health Sciences University, Dr George Mukhari Academic Hospital, Pretoria, South Africa

**Keywords:** Extraocular, Sebaceous, Muir-Torre Syndrome

## Abstract

**Background:**

Sebaceous carcinoma (SC) is a rare aggressive cutaneous malignant tumour. It accounts for less than 1 % of all cutaneous malignant tumours. Sebaceous carcinomas are divided into ocular and extraocular constituting 75 % and 25 % respectively. The most common extraocular site is parotid gland. Chest wall is a rare site of this tumour.

**Case presentation:**

In line with SCARE criteria (Agha et al., 2018 [1]), this report is a case of a 45-year-old African male patient who presented with a two-year history of a large right anterior chest wall tumour. He reported that the tumour started as a small lump, which grew gradually over a two-year period. The patient reported no history of visceral malignancy and radiation exposure. However, his risk factors were age and immunosuppression in the form of Human Immunodeficiency Virus (HIV). The tumour was initially diagnosed as sebaceous adenocarcinoma by incisional biopsy before the patient had been referred to the treating team a year before. Excision of the tumour with wide margins was undertaken, and histology results confirmed sebaceous carcinoma.

**Discussion:**

Sebaceous carcinoma is a rare and aggressive adnexal tumour. It is categorized as ocular and extraocular. Of these two, the more common is extraocular. The chest wall is a rare site for this tumour. The definitive diagnosis of this kind of tumour is done by tissue biopsy. In dealing with this tumour, surgical excision with wide margins is the standard treatment.

**Conclusion:**

Sebaceous carcinoma is a rare aggressive malignant tumour originating from sebaceous gland. Early diagnosis and wide excision with negative margins improves survival.

## Highlights

• Sebaceous carcinoma is a rare adnexal tumour.
• It occurs sporadically or in association with Muir-Torre Syndrome (MTS).
• MTS is a rare genetic disorder that predisposes patients to visceral and skin malignancies.
• Chest wall is rare site of sebaceous carcinoma.
• Surgical excision with wide margins is the standard treatment.

## 1 Background

Sebaceous carcinoma is a rare skin cancer derived from the epithelium of the sebaceous gland [2]. It is predominantly found on the periocular region and occurs commonly in Asian women [3]. It is categorized into ocular and extraocular. The common site is ocular, which, accounts for 75 % of cases [3]. Sebaceous carcinomas (SCs) form less than 1 % of all cutaneous malignancies [3]. It may occur sporadically or be associated with Muir-Torre Syndrome (MTS), which is a familial autosomal inherited disorder. Extraocular sebaceous carcinomas are thought to be uncommon and less aggressive than ocular counterparts [4].

## 2 Case presentation

**Fig. 1:**
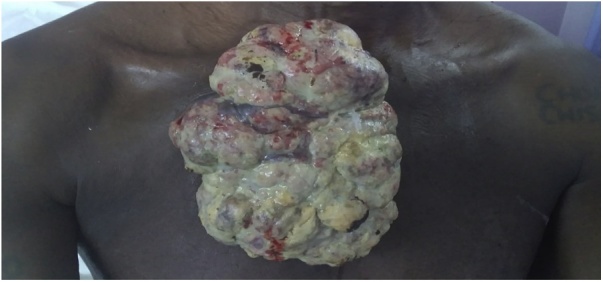
A: Pre-operative Image showing large ulcerated, multi-lobulated and mushrooming tumour.

The patient is a 45-year-old African male who presented with a two-year history of small lump on the right anterior chest wall. He reported that it was painless, firm and covered by hyper-pigmented skin. He sought for medical assistance after a year of noticing a gradual growth of the lesion. Subsequently, an incisional biopsy was done and histology results confirmed sebaceous adenocarcinoma. The patient absconded and presented a year later with a very large, pedunculated, mushrooming and fungating tumour. Its stalk was on the second intercostal space extending to the parasternal area (Fig. 1 A). The risk factors of this patient were age (45 years) and Human Immunodeficiency Virus; that is to say, he was HIV positive. On local examination, the tumour was found on the right anterior chest wall, with short and broad stalk on the second intercostal space and parasternal area. It had a mushroom configuration, which on long axis was extending from suprasternal notch to the lower third of sternal body. Transverse extend of the tumor was from right mid-clavicular line up to left parasternal area. The tumour was multi-lobulated, firm, non-tender, fixed to chest wall and had hyper-vascular, with sloughing areas. It was measuring 180 × 140 × 30 mm. There was no associated regional lymphadenopathy on examination. Examination findings of the other systems were normal.

**Fig. 2:**
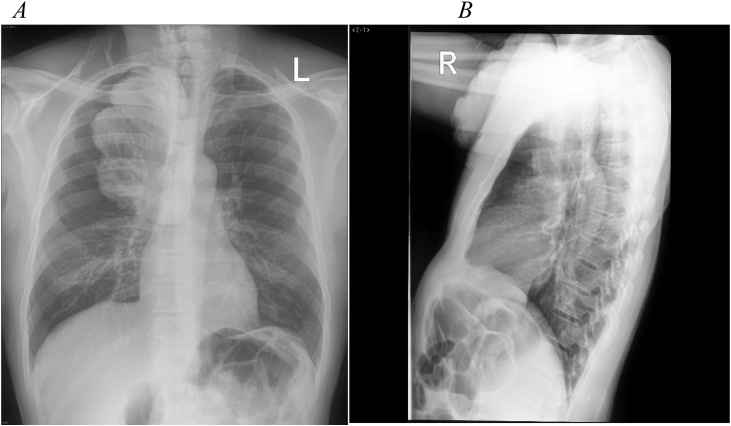
A: PA view showing multi-lobulated homogenous soft tissue density in the upper mediastinum, which extends to the right hemithorax. The rest of mediastinal structures look normal. B: lateral view showing multi-lobulated soft tissue homogenous overlying anterior chest wall. Opacity extends from thoracic inlet to superior part of gladiolus with suspicious intrathoracic extension via thoracic inlet.

Postero-anterior (PA) and lateral chest x-rays (CXRs) were done, with a PA showing multi-lobulated soft tissue homogeneous opacity in the upper mediastinum, which extends to the right hemithorax (Fig. 2A). A lateral view showed anterior chest wall homogeneous opacity extending from thoracic inlet up to the level of sterno-manubrial joint (Fig. 2B).

**Fig. 3:**
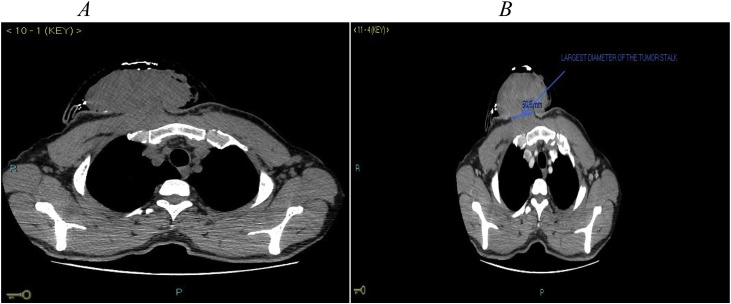
A: Axial view of non-contrast CT-scan showing a tumour with calcified capsule and with no evidence of bony erosion. B: Axial view of a contrast CT-scan showing multi-dense tumour with a broad stalk and loss of subcutaneous plane at the insertion of the stalk.

**Fig. 4:**
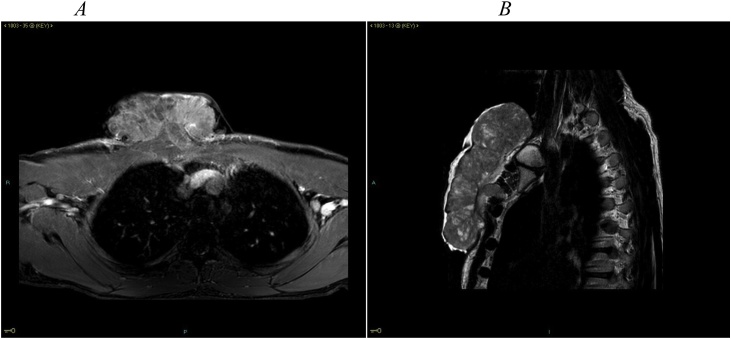
A: T1W axial view showing an expholytic tumour enhanced on contrast, with broad and short stalk attached and invading the right pectoralis major muscle. B: T2W para-sagittal view showing an expholytic tumour that is extending from thoracic inlet to the upper third of the sternal body, with a stalk that invades and attaches to the pectoralis major muscle.

A non-contrast computed tomography (CT) scan showed anterior chest wall tumour with a calcified capsule without bones involvement (Fig. 3A). A CT-scan with contrast on axial cuts showed a large tumour, with a broad stalk. The tumour was infiltrating the right pectoralis muscle, as evidenced by loss of subcutaneous plane between the tumour and the muscle (Fig. 3B). A type 2-weighted image (T2W) axial magnetic resonance imaging (MRI) view with contrast showed a hyper-intense tumour (Fig. 4A). The broad and short stalk was also demonstrated, invading the right pectoralis muscle. A T2W sagittal view showed that a tumour was extending superiorly up to thoracic inlet and inferiorly up to superior third of sternal body (Fig. 4B).

**Fig. 5:**
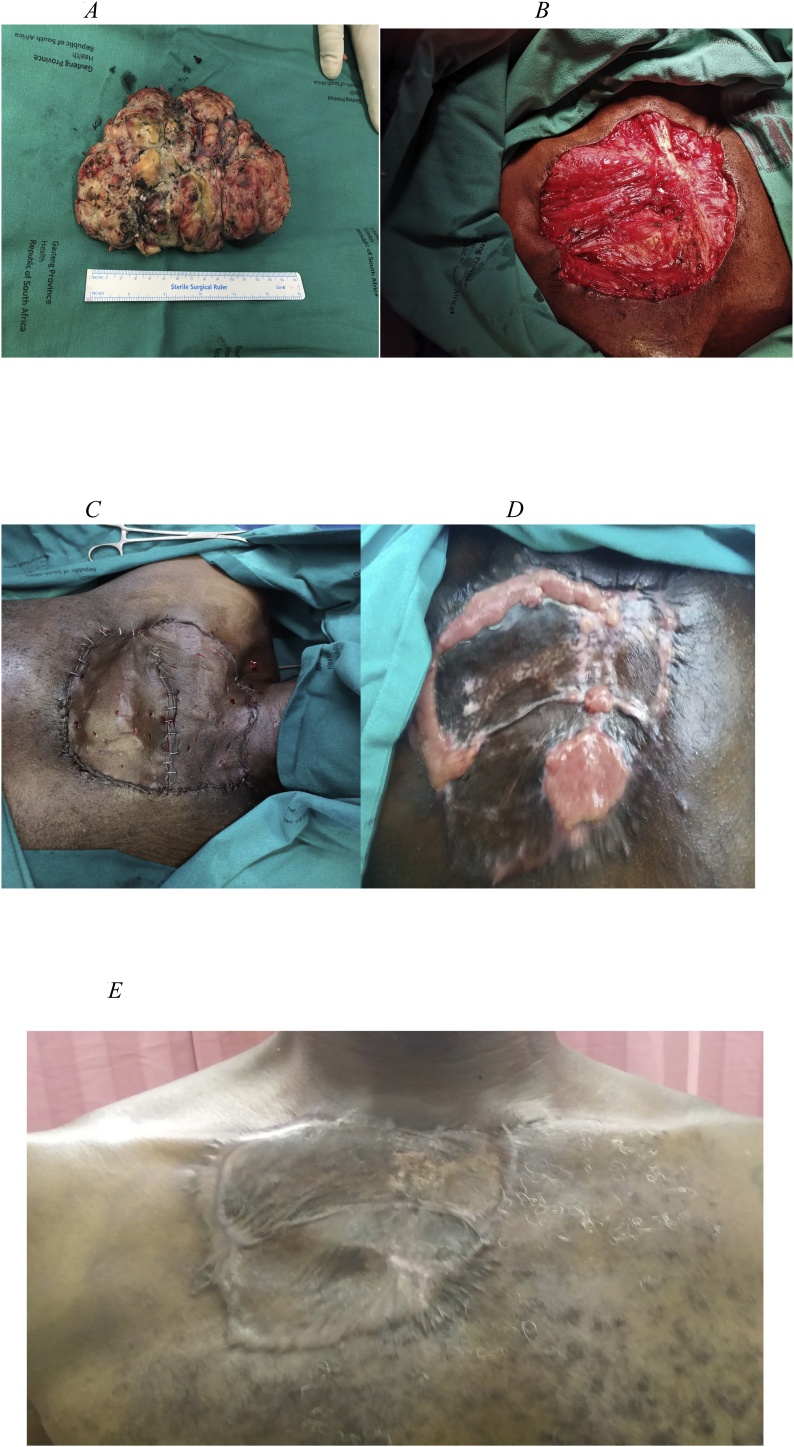
A: Tumour after excision measuring 140 × 180 × 30 mm. B: Tumour was excised with part of the right pectoralis major muscle. C: Skin graft undertaken to close the defect. D: Skin graft with areas of over-granulation. E: Final result with the defect closure.

Total excision of the tumour was undertaken with 1.5−2 cm wide surgical margins, both peripheral and deep. The invaded part of right pectoralis major muscle was also resected *en bloc* (Fig. 5A, B). A frozen section confirmed negative margins and the defect was dressed with a vacuum assisted closure (VAC) dressing to reduce wound margins and encourage uniform granulation of uneven wound surface. The skin graft was deferred pending definitive histology results for diagnosis. The histopathology results confirmed total tumour excision, and a skin graft was undertaken a week later (Fig. 5C). The skin graft complicated with severe over-granulation (Fig. 5D) for which small incisional biopsy was taken, which proved no recurrence. Conservative treatment was undertaken for a month, and the skin graft healed (Fig. 5E).

**Fig. 6:**
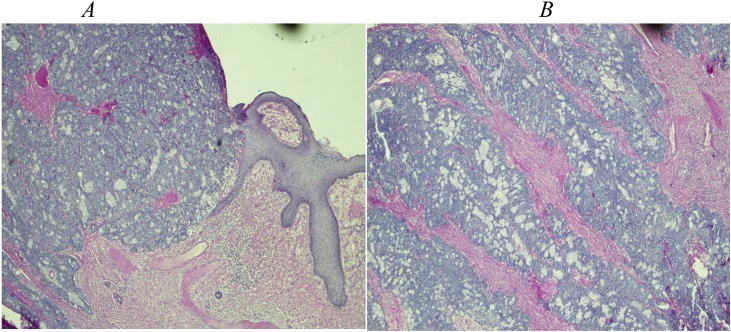
A: H/E section shows ulcerated skin with an invasive carcinoma with sebaceous differentiation. B: H/E (intermediate view) show basaloid tumour cells with cytological atypia and admixed with foamy/vacuolated cytoplasm in keeping with sebaceous cells.

Grossly, a 180 × 140 × 40 mm ulcerated and multi-lobulated tumour was received. On cut section, it was white-tan and firm. The hematoxy-eosin (H/E) stained sections showed ulcerated invasive sebaceous carcinoma evidenced by ulcerated skin with an invasive basaloid tumour (Fig. 6A) with sebaceous differentiated (Fig. 6B). The tumour cells were positive for epithelial membrane antigen (EMA) and p63 (some of basaloid cells) while B-cell lymphoma 2 (Bcl-2) and carcinoembryonic antigen (CEA) were negative. Excision was completed.

## 3 Discussion

Sebaceous carcinoma is an uncommon aggressive adnexal tumour. The great majority of SCs (75 %) occur in the periocular area, commonly, the eyelid [5]. Extraocular tumours account for only 25 % of sebaceous tumours [5]. The tumours occur sporadically or in association with Muir-Torre syndrome (MTS). MTS is a rare genetic condition that predisposes individuals to skin and visceral malignancies [6]. MTS is an autosomal dominant inheritance disorder. The risk factors for development of SCs include: age, Asian ethnicity, history of radiation on the head and neck, MTS and immusupression [7]. The other possible factors included in the literature are the use of the diuretics and infection by Human Papillomavirus (HPV) [7]. The loss of control of immune system to an oncogenic virus in HIV patient is a risk for development of SCs [8]. Nonetheless, one cannot deny the relevance of HIV-induced immunosuppression in the aetiology of these neoplasms [8]. Most of the literature indicates that SCs has female predominance and it commonly occurs at age range of 45–72 years [5]. A varied morphologic spectrum includes: basaloid, squamoid, organoid and neuro-endocrine [6]. This variable morphology poses a diagnostic dilemma in most cases [6].

The morphological subtype in this case report is basaloid. This SC subtype can mimic different types of neoplasms, which makes correct diagnosis a challenge [9]. The morphologic variation occurs more in extraocular than ocular sebaceous carcinoma. Pathogenesis of SC is unknown, but is thought to be due to inactivation of the cell cycle mediator tumour protein 21(P21) [2].

The clinical presentation is not a pathognomonic and is often non-specific. The tumour is usually firm; slow enlarging, yellowish to red-brown plaque, friable, crusted and often ulcerated. The size of extraocular tumour varies, with ranges of 6−10 cm on its greatest dimension. Judged against studies prior to this one, this tumour is the largest with the size of 180 × 140 × 30 mm to be reported.

The definitive diagnosis of this tumor is by histology results. The presence of sebocytes characterized by multi-vacuolated cytoplasm and pleomorphic nuclei suggests the diagnosis of SCs on microscopy [6]. However, immunostains are needed to confirm diagnosis because of the variations. EMA stain shows sebaceous differentiation [6].

Adjunct investigations include the CXR, CT-scan and MRI, as done in this case report. The MRI is an important modality not only for diagnosis, but also for planning of surgery. It gives adequate information about location, size and extent of invasion of adjacent structures by the tumour. Surgical excision with wide margins is the standard treatment [3]. In case of regional lymph node or distant metastases, radiotherapy used alone or with chemotherapy may reduce morbidity [3].

On both physical examination and radiological investigations, the patient did not have regional lymph node involvement. This implies a better diagnosis. The rate of metastasis in sebaceous tumour is about 14–25 %, which is similar for both extraocular and ocular types [6]. The nodal involvement is approximately 8–12 % [3]. The local recurrence is about 9–36 % post-operatively [3]. This patient was followed up for four months with no signs of recurrence; however, one should be aware that recurrence has even been reported after five years; hence, follow-up is vitally important.

## 4 Conclusion

Sebaceous carcinoma is a rare but aggressive tumour, which is notorious for mimicking other tumours. The earlier the diagnosis, the better the prognosis. In the case of this tumour, histological analysis is the gold standard for diagnosis. Surgical excision with wide margin is standard treatment. A recurrence is uncommon, hence follow-up is mandatory.

## Funding

None.

## Ethical approval

Sefako Makgatho University Research Ethics Committee (SMUREC) approved the publication of this case report.

**SMUREC/M/287/2019**

INSTITUTIONAL REVIEW BOARD (IRB00010386).

## Consent

Written informed consent to publish this case report and images was obtained from the patient. The copy of written informed consent will be available for Editor-in- Chief on request.

## Author contribution

Dr. Sekgololo J.M. wrote the case report.

Prof Chauke R.F. and Dr. Tshazi N., both critically revised the case report.

## Registration of research studies

Not applicable, because is a single case report.

## Guarantor

Dr. Sekgololo J.M.

## Provenance and peer review

Not commissioned, externally peer-reviewed.

## Declaration of Competing Interest

None.
